# Genetics in Keratoconus – What is New?

**DOI:** 10.2174/1874364101711010201

**Published:** 2017-07-31

**Authors:** Sarah Moussa, Günther Grabner, Josef Ruckhofer, Marie Dietrich, Herbert Reitsamer

**Affiliations:** Paracelsus Medical University Salzburg, Department of Ophthalmology and Optometry, Müllner Hauptstr. 48, 5020 Salzburg, Austria

**Keywords:** Keratoconus, Genetics, Environmental risk factors, Non-inflammatory, Irregular astigmatism, Genomic loci

## Abstract

**Background:**

Keratoconus is characterized as a bilateral, progressive, non-inflammatory thinning of the cornea resulting in blurred vision due to irregular astigmatism. Keratoconus has a multifactorial etiology, with multiple genetic and environmental components contributing to the disease pathophysiology. Several genomic loci and genes have been identified that highlight the complex molecular etiology of this disease.

**Conclusion:**

The review focuses on current knowledge of these genetic risk factors associated with keratoconus.

## 1 INTRODUCTION

Keratoconus (KC), comes from the Greek words keras (cornea) and konos (cone). It was first described in literature in 1854 (Nottingham). KC is characterized as a (mostly) bilateral, progressive, non inflammatory thinning of the cornea resulting in blurred vision due to irregular astigmatism. Keratoconus usually manifests itself in the second decade of life with a descending progression after the patient reached the third decade. Each step of the pathogenethic process has been studied quite thoroughly over the last decades. It has been shown that - beside genetic factors - environmental effects such as inflammation, eye rubbing, allergic eye disease and wearing of contactlenses can play a role in the development of KC. Despite of these detailed research efforts the etiology of KC is still poorly understood [1].

The main challenge remains to distinguish between association, cause and effect [2].

Usually the definition of KC includes the notion of a noninflammatory process [3, 1]. However, recent studies refute this theory as there is evidence of overexpression of inflammatory mediators such as cytokines and interleukin 6 (IL-6) in tears of KC patients [4, 5] and inflammation is currently considered by some researchers to play an at least contributing role in the pathogenesis of KC [6] to add to genetic and environmental factors [7]. This article offers a review of the current knowledge on genetic aspects of keratoconus.

## 2 SIGNS AND SYMPTOMS

KC is presenting with variable clinical signs. In moderate stages a very common slit lamp sign is Fleischer’s ring located in the basal epithelium around the cone which can be partial or complete. It consists of iron deposits [8]. Other clinical slit lamp characteristics are Vogt’s striae, which are fine vertical lines due to compression of Descemet’s membrane [8]. In advanced cases patients may develop an *“acute”* KC, leading to breaks in Descemet’s membrane leading to a “hydrops” causing stromal edema, vision loss, and associated pain. For patients who wear contact lenses, corneal scarring is a very common feature [9].

The most sensitive diagnostic tool to detect keratoconus even in early stages is corneal tomography even if there are no further symptoms or signs.

KC affects both men and women. However, it remains unclear whether men or women have higher prevalence of KC. The majority of recent papers published after 1970s [8] indicate a certain preponderance of men over women with KC while other studies published prior to 1970s and two recent studies reported the opposite [8].

## 3 TREATMENT

The first aim in treating a patient with KC is to correct vision to the possible maximum. In early stages spectacle correction is usually satisfactory. In mild or moderate stages presenting with irregular astigmatism, the treatment of choice are *contact lenses*, especially rigid gas permeable ones. If contact lenses are not tolerated *Intra Corneal Rings* (ICR, different brands available) could be a solution to enable spectacle correction. Since the late nineties collagen cross-linking (CXL) has been proven to be successful in “stiffening” the cornea thereby arresting progression and flattening the cornea by preventing enzymatic degradation of stromal collagen – at least for the observation time available by now and for a percentage of patients followed for these extended periods of time that are still short compared to the patients life expectancy [10, 11].

However, sometimes patients with advanced and severe KC cannot tolerate or improve their vision sufficiently with contact lenses, ICRs or CXL and will eventually need surgery. The traditional surgical intervention has been penetrating keratoplasty, now mostly replaced by deep lamellar keratoplasty (DLK). Further research will significantly improve our understanding of the disease and further our therapeutic options.

## 4 RISK FACTORS FOR KC

There is evidence that the etiology of KC is multifactorial and that environmental factors play an important role. They are supposed to have a trigger role in genetically predisposed individuals [8].

- Environmental factors, which have been recognized, are: eye rubbing, atopy and UV exposure, although the relative contribution of all these factors is currently unknown [12].The theory goes that environmental factors are causing oxidative stress to KC corneas and, because of the inability of KC corneas to process reactive oxygen species (ROS), a degradation process is initiated that leads to corneal thinning and loss of vision [13] due to a lack of corneal enzymes such as aldehyde dehydrogenase class 3 (ALDH3), catalase, or superoxide dismutase to remove or neutralize the ROS [14].
- Eye Rubbing - An association has been reported between eye rubbing and KC [15]. It is interesting that in cases of asymmetric KC it is the most affected eye which was rubbed most vigorously [16, 17]. The mechanism may be that mechanical stress is causing a microtrauma leading to elevated levels of matrix metalloproteinases MMP-1 and MMP-13. These factors may lead to progrssion of KC associated with an apoptosis of keratocytes [18, 19].
- Atopy - Studies show that allergy against pollen, dust, antibiotics or animal fur, can be associated with KC compared to controls or the general population [20-22]. Bawazeer *et al.* concluded that atopy was not significantly associated with KC but rather with the eye rubbing caused by the itch of the condition [15].
- Ultraviolet light (UV) – It is a source of reactive oxygen species (ROS) and it is assumed that excessive exposure to sunlight leads to oxidative damage in KC corneas, in which there is a reduced amount of the enzymes including aldehyde dehydrogenase class 3 (ALDH3) and superoxide dismutase necessary to remove the ROS [23, 14]. As studies showed that there is a higher prevalence of KC in countries with a hotter climate compared to Europe and North America there is belief that the high sun exposure in these countries accounts for the high prevalence [8]. Nevertheless, it is likely that the oxidative damage caused by UV radiations combined with a genetic factor such as consanguinity precipitates or accelerates the disease process [8]. An experimental finding, that mice exposed to UV light demonstrate a degeneration of stromal collagen and stromal thinning with a marked loss of keratocytes adds to the theory [24].

## 5 GENETICS IN KC

### 5.1 KC in Families

Although the most frequent type of KC is sporadic [1], many studies have reported the presence of large number of familial KC with strong evidence for familial aggregation of KC collected. The majority of familial keratoconus is inherited through an *autosomal dominant pattern.* Other models of inheritance such as autosomal recessive pattern have been suggested especially in populations of high consanguinity. However most cases of KC appear to be sporadic in a polygenic fashion [24-27] First degree family members are at higher risk than normal population. This supports a genetic effect on developing KC [28]. 10% of these patients showed a positive family history [29]. Twin studies have emerged as a powerful tool to determine the effect of heredity on disease manifestation. A small study demonstrated higher concordance of keratoconus in monozygotic than in dizygotic twins, with a greater similarity of phenotype in the monozygotic twins, consistent with a strong genetic component to this disorder. Gordon-Shaag et al stated that children of consanguineous parents had a fourfold risk of KC compared with children of unrelated parents and this association was much stronger with parents married to first cousins than second cousins [30]. This result was further confirmed in a similar study conducted with students from an Arab College in Haifa in which a fivefold risk of KC [31] was found. If father and mother are cousins in first line, they could both be carriers of a mutant allele at the same locus leading to corneal ectasia. It is interesting that these studies rather point to an autosomal recessive inheritance. This is clearly in contrast to KC in a positive family history in western families described in the literature, where family pedigree suggesting an autosomal dominant inheritance [1, 8, 32]. Whatever genetic transmission is infered, the much higher risk of KC in first-degree relatives compared with the general population indicate a strong genetic component to KC.

## 5.2 Linkage Analysis

Complex genetic phenomena and interaction make the identification of the causative genes difficult. One of the strategies to localise the contributing gene(s) is linkage analysis [32].

In a linkage study multiple families, pedigrees in more than one generation are studied. Affected and unaffected members undergo genetic analysis. Chromosomal regions that are distributed equally between affected and unaffected members are not likely to be the causative genes. After defining the chromosomal regions, the mapped gene with that region gets determined.

One of the disadvantages of linkage analysis is the possibility of “phenocopies” and reduced penetrance in any given KC pedigree. The inclusion of individuals with simple astigmatism, thin corneas without ectasia, or borderline (forme fruste) KC disease is also controversial [33]. Another point in case is that despite the typical age of KC onset in the second decade of life, onset has been reported over the age of 50 years [34]. Therefore the classification of individuals as being ”unaffected” is difficult. Finally, different loci and genes may be implicated in families of different ethnicities, confounding a replication of the loci. But there is high hope that progress will be made using recent advances in next-generation sequencing technologies [35]. Family-based linkage studies have identified at least 19 candidate genetic loci that may harbour genetic mutations for KC [8]. This means that KC could be caused by *mutations in a number of different genes in different families.* While most of these loci have not been independently replicated, the 5q21.2 locus has been independently identified in three different studies [27, 36, 26]. This region has been further confirmed with high density single nucleotide polymorphisms (SNPs) based linkage [37]. The overlapping region from these three studies strongly suggests the possibility of a common locus for KC pathogenesis. Bisceglia et al reported another linkage locus chr5q32-33 which was also identified as suggestive linkage with KC by Li *et al.* [27, 36]. A suggestive linkage locus in chr14q11.2 was suggested by these two studies. Tyynismaa *et al.* identified locus chr16q22.3-q23.1 who is very close to a suggestive linkage region identified by Bisceglia *et al.* [26, 27].

Similar to other complex diseases, gene-gene interaction is another defining genetic event that should be considered. How different genes under the effect of environmental factors interact with each other to form the subsequent phenotype has not yet been studied in sufficient detail. It should be noted that Burdon *et al.* reported two genomic regions chr1p36.23-36.21 and chr8q13.1-q21.11 with equal evidence of linkage (LOD score of 1.9 each) [38]. They also stated that single locus analysis resulted in less LOD score (logarithm (base 10) of odds) than analyzing two regions concurrently and this finding strengthens the assumption of these important interactions.

These complexities in interpreting gene analyses have restricted defining new loci and were partly responsible for the unproductive investigation in finding responsible gene(s) for gene therapy in keratoconus [32].

In the last years a high number of candidate genes have been studied in relation to KC pathogenesis. We will focus on VSX1 (visual system homeobox 1), MIR184 (microRNA 184), and DOCK9 (dedicator of cytokinesis 9) and SOD1 in addition to other candidate single nucleotide polymorphisms (SNPs) in other genetic loci.

## 5.3 Candidates Genes Associated With KC

As the identification of genetic risk factors due to the complex etiology of KC is complicated, the identification of genetic componenents in families with suspected dominant forms of KC, has been employed to study KC cohorts. “Candidate gene” approaches are useful to study multifactorial complex diseases and enables researchers to identify even small gene effects using large case-control cohorts. Potential candidate genes in KC are those associated with other corneal dystrophies, connective tissue disorders or located on chromosomes where chromosomal aneuploidy or breakpoints are associated with the disease, for example, chromosome 21 in Down syndrome [39-42].

## 5.4 VSX1 (Visual System Homeobox 1)

VSX1 is located within a linkage locus on chromosome 20p11–q11. This locus is known for a corneal dystrophy called posterior polymorphous dystrophy (PPCD) [43-45]. It has been shown that PPCD and KC have a similar corneal curvature. In both pathologies the posterior surface of the cornea, and here especially Descemet’s membrane, plays a role. PPCD and KC might be linked due to poor case defnition. VSX1 encodes a pairlike homeodomain transcription factors family that plays a role in cranofacial and ocular development [46, 47]. It is expressed by keratocytes in injured corneas and plays a role in fibroplastic transformation [48]. Since the first report by Heon and colleagues [44] numerous studies have evaluated the association of variants in VSX1 and keratoconus [45-52]. Another significant association in Han Chinese population was found in two synonymous (rs56157240 and rs12480307) and one missense tag SNP (rs6050307) in the VSX1 gene [53]. Two heterozygous mutations (N151S and G160V) and an intragenic polymorphism were significantly associated with increased risk of KC in Korean patients [54]. One novel missense heterozygotous change (p.Leu268His) was identified in five KC patients from two unrelated families [55]. In Saudi cohorts it failed to show any association [56]. The expression of VSX1 in human or mouse cornea remains unclear since many studies did not confrm the expression in cornea [41, 57, 58]. Mouse models with the loss of VSX1 function did not show cornea-related phenotypes [46] Since the original report in 2002, many studies have examined the potential. Most of the identifed variants are polymorphic [59]. It remains unclear whether VSX1 mutations contribute to the pathogenesis of KC [8, 60, 61]. It is possible that mutations in VSX1 only affect a very small percentage of KC patients, which is consistent with the concept of genetic heterogenity of KC. It seems more and more plausible that VSX1 may not play a significant role in the pathogenesis of KC. Future research efforts focusing on the identification of novel genetic factors in KC are needed.

## 5.5 MiR-184

miR-184 is a microRNA, which are small regulatory strands of RNA with 19-25 nucleotides in length. They bind to complementary sequences mostly in the 3′ untranslated region (UTR) of mRNA of target genes and lead to mRNA degradation or translational suppression. A 5Mb genomic region on chr15q22-q25 was originally mapped in a large three-generation Northern Irish family with 18 affected individuals. All the affected family members had severe anterior KC and early-onset anterior polar cataract [24]. The inheritance was autosomal dominant. To establish whether mutations in mir-184 were associated with KC, mir-184 was subsequently screened in a cohort of 780 KC patients. Rare variants were identified in two (0.25%) patients, but the variants did not fully segregate with disease, suggesting that mir-184 variants are not a common cause of isolated KC [62]. More research again will be necessary to study whether miR-184 may regulate the expression of other KC candidate genes.

## 5.6 DOCK9

Another linkage analysis identified another potentially pathogenic variant - DOCK9. DOCK9 (Dedicator of cytokinesis 9) (OMIM 607325) – that encodes a member of the DOCK protein family which possesses GTP/GDP exchange factor activity and specifically activates G-protein CDC42 (57). This locus was found in a large dominant KC family of Ecuadorian origin. A mutation screening of eight candidate genes within the 13q32 locus identified 100% segregation of one non-synonymous mutation and three different sequence variants in the DOCK9 gene and two additional genes, IPO5 (importin 5, OMIM 602008) and STK24 (serine/threonine kinase 24, OMIM 604984). All these three genes are expressed in the human cornea [63]. However, this finding still requires to be replicated in other KC families and patients [63, 64].

## 5.7 SOD1

SOD1 (Superoxide dismutase 1) encodes a major cytoplasmic antioxidant enzyme that metabolizes superoxide radicals and provides a defence against oxygen toxicity [65]. Since oxidative stress has been hypothesized to play a role in the etiology of KC [66, 67] and given the association of trisomy 21 (Down syndrome) with KC, association of variants in SOD1 gene localized on chromosome 21 has also been investigated. SOD1 has been selected as a candidate gene and examined in many KC-related studies [48, 67, 49, 68-71]. However, no mutations in SOD1 have been identifed in KC patients. It remains undetermined whether SOD1 plays a role in the pathogenesis of KC.

## 5.8 Genome-Wide Association Studies

Genome-wideassociation studies (GWAS) examine several hundred thousand to over a million SNPs in hundreds to thousands of individuals using high throughput DNA genotyping technology to provide a powerful platform to identify common risk variants in complex genetic disease [72-74].

It has been shown that GWAS is a powerful tool to investigate the genetic etiology of many complex traits and diseases, including Fuchs’ corneal dystrophies (FECD) and central corneal thickness [75, 65].

There have been a number GWAS reporting the association of CCT with sequence variants near or within many genes, including ZNF469, COL5A1, RXRA- COL5A1, COL8A2, AKAP13, AVGR8, FOXO1, FNDC3B, TJP1, NR3C2, LRRK1, FDF9-SGCG, LCN12-PTGDS, ADAMTS6, CHSY1, HS3ST3B1-PMP22, GLT8D2, SMAD3, VKORC1L1, COL4A3, FAM46A-IBTK, LPAR1, ARID5B, TBL1XR1- KCNMB2, ARHGAP20-POU2AF1, C7ORF42, MPDZ-NF1B, USP37, GPR15, TIPARP and WNT10A [75-81].

Two CCT-associated genomic regions (FOXO1 and FNDC3B) have been associated with KC risk. These findings showed that collagen and extracellular matrix pathways play a role in the regulation of CCT and potentially KC [78].

Cuellar-Partida showed that WNT10A increases the risk for KC twice [80].

In another two-stage genome wide linkage scan in KC families Li and collegues identified a locus at chromosome 5q23.2, overlapping the gene encoding lysyl oxidase (LOX, OMIM 153455) with LOX being involved in corneal collagen and elastin crosslinking [82].

Dudakova *et al.* recently stated that a locus within LOX has a protective effect against KC [83].

Two other studies identifed missense variants in ZNF469 in 12.5% and 23.3% of sporadic KC patients in UK/Switzerland and New Zealand highlighting the potential role of ZNF469 in the development of KC [84, 85]. Karolak *et al.* could not find a significant enrichment of sequence variants in ZNF469 in Polish patients with KC [86] Li et al reported the first GWAS in 2011 in a Caucasian population of 222 patients and 3324 controls [82]. No genome-wide significant association was found but they identified a suggestive association with a genomic region located near RAB3GAP1 (RAB3 GTPase activating protein subunit 1) gene on chromosome 2q21.3. Bae et al could also replicate this association. This region could play a role in KC susceptibility.

Addidionally RAB3GAP1 is also associated with Warburg Micro Syndrome which is a rare autosomal recessive syndrome with ocular defects, such as microphthalmos, microcornea, congenital cataracts, and optic atrophy [87-90].

Burdon *et al.* carried out the second GWAS with KC. Using GWAS they identified a SNP (rs3735520) at the HGF (OMIM 142409) locus to be associated with KC in cohorts from Australia, USA and Northern Ireland (90). No variants showed genome-wide significance. This association has been independently replicated by Sahebjada *et al.* [91]. Dudakova *et al.* also stated that HGF is associated with a higher risk in developing KC [92]. HGF has also been associated with refractive error in several populations [93-95] and suggests the potential involvement of HGF-related inflammatory pathways.

## CONCLUSION

KC is the most common ectatic disorder of the cornea. It usually affects people of both genders and all ethnicities in the second decade of life with a descending progression after thirties. Genetic and environmental factors play a major role in the pathogenesis of KC. However, the etiology of keratoconus is far from understood, with environmental, behavioral, and genetic factors all - in a variety of ways - contributing to the disease. Newly developed genetic technologies including whole-exome or genome sequencing and GWAS will significantly propel the genetic research of keratoconus, which in turn will improve our understanding of the genetic factors in the etiology of keratoconus. Hopefully this will ameliorate our diagnostics and allow for targeted treatments.
